# Genotype-Phenotype Correlations Identify Phenotypic Differences in Sarcomere Mutation-Positive Hypertrophic Cardiomyopathy in the NHLBI HCM Registry

**DOI:** 10.64898/2026.07.28.26359171

**Published:** 2026-07-30

**Authors:** Jonathan H. Chan, Christopher Grace, Kate Thomson, Dong-Yun Kim, Patrice Desvigne-Nickens, Paul Kolm, John P. DiMarco, Milind Desai, Raymond Y. Kwong, Carolyn Y. Ho, William Weintraub, Stefan Neubauer, Christopher M. Kramer, Hugh Watkins, Anuj Goel

## Abstract

**Background:**

Previous studies of genotype-phenotype correlations in hypertrophic cardiomyopathy (HCM) have presented inconsistent conclusions, potentially reflecting small studies and non-uniform phenotyping.

**Objectives:**

We aimed to identify genotype-phenotype correlations in sarcomeric variant-positive HCM cardiac magnetic resonance (CMR) imaging and adverse outcome data in the National Heart, Lung, and Blood Institute (NHLBI) HCM Registry.

**Methods:**

Of 2750 overall patients, 915 sarcomeric variant-positive ones were subgrouped by genotype. CMR measures of left ventricular (LV) hypertrophy, fibrosis and function, and adverse events, were compared. Findings were meta-analyzed with prior studies from systematic review.

**Results:**

Patients with pathogenic variants in thick-filament genes had greater hypertrophy than those in thin-filament genes - maximal LV wall thickness (maxLVWT) (21.7 ± 5.0 vs. 20.0 ± 4.4mm, *P* <0.01) and indexed LV mass (81.9 ± 26.0 vs. 71.7 ± 13.3g/m ^2^ , *P* <0.001). Of the former, *MYBPC3* carriers had greater maxLVWT than *MYH7* carriers (22.2 ± 5.2 vs. 20.9 ± 4.5mm, *P* <0.01) but lower LV ejection fraction (63.1 ± 8.4 vs. 65.3 ± 8.4%, *P* <0.001). Findings remained significant after covariate adjustment and meta-analysis. 23 (∼1%) patients had >1 disease-linked variants with only 3 (∼0.11%) carrying >1 pathogenic variants. *MYBPC3* carriers had lower risk of a multiple event composite than *MYH7* carriers.

**Conclusions:**

In the largest CMR-based study to date, we identified significant phenotypic differences that characterize disease-gene subgroups and resolved prior discrepancies through systematic review and meta-analysis. However, carriage of >1 sarcomeric variants did not contribute much to variation in phenotypic severity in the general HCM population due to its rarity.

**Condensed Abstract:**

Prior studies of genotype-phenotype correlations in hypertrophic cardiomyopathy have produced inconsistent findings. In the largest study to date with CMR imaging, we identified significant correlations between disease phenotypes and genotype subgroups of sarcomeric variant-positive patients from the NHLBI Hypertrophic Cardiomyopathy Registry. Patients with disease-linked *MYBPC3* variants had greater hypertrophy and lower ejection fraction than those with *MYH7* variants but also less incidence of clinical outcomes. Patients with multiple variants were strikingly rare (∼1% of all patients). Ultimately, we discovered phenotypic differences that characterize rare genotype subgroups and resolved prior discrepancies through systematic review and meta-analysis.

## Full Text Availability

The license terms selected by the author(s) for this preprint version do not permit archiving in PMC. The full text is available from the preprint server.

